# Water Extract of Portulaca Oleracea Inhibits PEDV Infection-Induced Pyrolysis by Caspase-1/GSDMD

**DOI:** 10.3390/cimb45120637

**Published:** 2023-12-18

**Authors:** Yu Zhang, Yueyue Liu, Shifa Yang, Bin Yin, Zengcheng Zhao, Zhongli Huang, Jiaqiang Wu, Shuqian Lin, Xin Wang

**Affiliations:** 1 College of Veterinary Medicine, Qingdao Agricultural University, No. 700 Changcheng Road, Qingdao 266109, China; 20212213032@stu.qau.edu.cn; 2 Institute of Poultry Science, Shandong Academy of Agricultural Science, Shandong Provincial Animal and Poultry Green Health Products Creation Engineering Laboratory, Jinan 250100, Chinayangshifa@saas.ac.cn (S.Y.); yb53650@163.com (B.Y.); wjiaqiang666@163.com (J.W.); 3 Key Laboratory of Animal Resistant Biology of Shandong, College of Life Sciences, Shandong Normal University, Jinan 250014, China

**Keywords:** PEDV, pyrolysis, caspase-1/GSDMD, *Portulaca oleracea*, in-vitro analysis

## Abstract

Porcine epidemic diarrhea virus (PEDV) belongs to the coronavirus family and the coronavirus genus, causing contact enteric infection in pigs. It is one of the most serious diseases that threatens the pig industry. However, there is currently no specific drug to prevent and treat the disease, indicating that we need to be vigilant about the spread of the disease and the development of anti-PEDV drugs. The dried aerial parts of the plant *Portulaca oleracea* in the family Portulacaceous, whose decoction can be used to treat acute enteritis, dysentery, diarrhea, and other diseases. This study explored the potential mechanism of water extract of *Portulaca oleracea* (WEPO) in PEDV-induced pyroptosis in Vero cells. PEDV decreased the viability of Vero cells in a dose- and time-dependent manner, causing cell damage, upregulating the level of intracellular Nlrp3, and inhibiting the level of Gasdermin D (GSDMD) and the activation of Caspase-1. WEPO can inhibit PEDV-induced pyroptosis, reduce the elevation of inflammatory factors caused by infection, and exhibit a dose-dependent effect. Knockdown of Caspase-1 and GSDMD separately can induce the production of the inflammatory factor IL-1β to significantly decrease and increase, respectively. These results suggest that WEPO can inhibit cell pyroptosis caused by PEDV and that the Caspase-1 and GSDMD pathways play an important role in this process.

## 1. Introduction

Porcine epidemic diarrhea (PED) is caused by the porcine epidemic diarrhea virus (PEDV) and manifests similarly to transmissible gastroenteritis (TGE) in swine, with symptoms including diarrhea, vomiting, anorexia, dehydration, and weight loss in piglets [1]. PEDV is a member of the genus Alphacoronavirus in the family Coronaviridae of the order Nidovirales. During outbreaks, the mortality rate of newborn piglets at the time of the outbreak has reached 100% [2], posing a serious threat to public food safety worldwide and causing serious effects on the development of the pig industry [3]. Currently, there is no specific drug available for the clinical treatment of PEDV, making it urgent to further investigate the pathogenesis of the virus and develop antiviral drugs.

After PEDV infects the host and is recognized by the pattern recognition receptor, it transmits signals to the downstream signaling pathway to produce inflammatory factors such as IL-1β and IL-6, ultimately completing the host’s immune defense process [4]. However, PEDV can also escape the host’s natural immune response, and escaping the natural immune defense is important in the pathogenesis of PEDV infection [5]. As an important component of the innate immune response, the inflammasome also plays a pivotal role in the defense against viral infection [6]. Studies have shown that PEDV infection is associated with cell damage and excessive inflammation, excessive activation of inflammatory factors may promote cell pyroptosis, and pGSDMD-mediated pyroptosis could inhibit PEDV replication [7,8]. Therefore, the inhibition of pyroptosis may present a novel clinical strategy against PEDV.

WEPO has been reported to inhibit the proliferation of PEDV, downregulate the expression of inflammatory factors in PEDV-infected cells, and inhibit NF-κB activation of the signaling pathway [9]. *Portulaca oleracea* belongs to the *Portulaceae* plant, which is a widely distributed medicinal and food-homologous plant. Many constituents of *Portulaca oleracea* have been isolated, including flavonoids, alkaloids, fatty acids, terpenoids, polysaccharides, vitamins, sterols, proteins, and minerals [10]. There are seven different flavonoids in *Portulaca oleracea*, including kaempferol, myricetin, luteolin, apigenin, quercetin, genistein, and genistin [11]. It was reported to treat acute enteritis, dysentery, and diarrhea in the Compendium of Materia Medica and other ancient books. Modern pharmacology also confirmed that *Portulaca oleracea* and some of its monomer components have certain protective effects on intestinal diseases, such as inflammatory bowel disease (IBD), irritable bowel syndrome (IBS), and ulcerative colitis (UC) [12]. When analyzing the active ingredient target of *Portulaca oleracea* with viral infection, enteritis, and other related pathways by using network pharmacology, it was found that NLRP3, caspases, IL-1β, and others play an important role in enteritis-related diseases, while IL-1β and caspases are related to pyroptosis. Based on this, this study aims to further investigate the role of WEPO in inhibiting PEDV infection through a stable infection mode in vitro, the role of WEPO in inhibiting PEDV replication and its impact on the Caspase-1/GSDMD pyroptotic pathway, followed by the role of Caspase-1/GSDMD in the inhibition of PEDV by WEPO. Additionally, the role of quercetin, one of the main components of purslane, in inhibiting the proliferation of PEDV and its pyroptosis pathway was also examined. This will further explore the mechanism of WEPO or its main components in inhibiting PEDV proliferation and provide new ideas for the prevention and treatment of PED using Chinese veterinary medicine.

## 2. Materials and Methods

### 2.1. Cell Lines and Virus

The Vero cells were cultivated in Dulbecco’s modified Eagle’s medium (DMEM; Gibco, Grand Island, NE, USA), which was supplemented with 10% heat-inactivated fetal bovine serum (FBS; Gibco, Grand Island, NE, USA). The cells were incubated at 37 °C in a 5% CO_2_ incubator. When the cells in the cell flask grow to 80–90%, the cells were divided into cell plates, cultured for 24 h, and WEPO or PEDV was added. PEDV (SX-1 strain) was isolated from diarrheal pigs in Shandong Province, China, and its TCID50 was measured after more than 24 passages at 10–7.67/mL.

### 2.2. CCK-8 Assay

In the experiment, we selected cells in the logarithmic growth phase for testing. After treating the Vero cells (infected with PEDV or added with WEPO) with CCK-8 (cell counting kit-8, from Dojindo Chemical Research Institute) for 3 h, we accurately measured their absorbance at 450 nm using a microplate reader (Hangzhou AllSheng Instrument Co., Ltd., Hangzhou, China). Afterward, we calculated the cell viability according to the instructions of the CCK-8 kit. It is worth noting that the blank wells only contain culture medium and CCK-8 reagent, and our experiment was repeated three times to ensure the reliability of the results.

### 2.3. Measurement of LDH Release and ATP Production

LDH release was employed to evaluate cell damage, and the supernatant of Vero cell cultures was collected at room temperature. LDH was then measured using an LDH detection kit according to the manufacturer’s instructions. ATP production was used to assess cell activity, and Vero cells were collected at room temperature. ATP was measured using the ATP Assay Kit according to the manufacturer’s instructions. The levels of LDH release and ATP production were calculated based on the instructions provided.

### 2.4. Reverse Transcription-Polymerase Chain Reaction (RT-PCR)

The mRNA expression of different cytokines in Vero cells was determined using RT-PCR. Briefly, total RNA was extracted from Vero cells using the SteadyPure Quick RNA Extraction Kit (catalog number AG21023, Accurate Biology, China). The extracted RNA was used for cDNA synthesis and served as a template for real-time PCR [13]. RT-PCR was carried out with the help of a SYBR Green Premix Pro Taq HS qPCR Kit.

The amplification reaction mixture was prepared by mixing 10 μL of 2X SYBR Green mix, a specific primer set (0.4 μL each), cDNA (2 μg), and DEPC-treated water to a total volume of 20 μL. The thermocycling conditions used were as follows: initial denaturation at 95 °C for 30 s, followed by 40 cycles of denaturation at 95 °C for 5 s, and extension at 58 °C for 30 s. GAPDH was used as an internal control, and mRNA expression levels were analyzed using the 2^−ΔΔCt^ method. The primers used are as follows: **IL-1β** (forward 5′-ACCATGGCCATAGTACCTGAACCCG-3′; reverse 5′-AATTAGGGAGAGAGGACTTCCAT-3′) **NLRP3** (forward 5′-TTGCTGCGATCAACAGGAGA-3′; reverse 5′-CGGTCCTATGTGCTCGTCAA-3′) **GSDMD** (forward 5′-TCGGGTCGCAGTTTCACTTT-3′; reverse 5′-ACCACATACACGTTGTCCCC-3′) **GADPH** (forward 5′-ACGGCCAGGTCATCACTATTG-3′; reverse 5′-AGGGGCCGGACTCATCGTA-3′) **PEDV** (forward 5′-CGCAAACGGGTGCCATTATC-3′; reverse 5′-CCTTGTTAGTGGGTACAGCGT-3′).

### 2.5. Immunofluorescence

Infected Vero cells were fixed with 4% paraformaldehyde for 20 min and washed three times with PBS. The cells were then incubated with 0.1% Triton X-100 for 15 min at room temperature and washed five times (3 min each time) with PBS. The cells were then blocked with 5% BSA for 30 min at room temperature and washed three times (3 min each time) with PBS. The primary antibody was added to the sample and incubated overnight at 4 °C. The primary antibody was removed, and the sample was washed five times (3 min each time) with PBS. The secondary antibody was then added, and the sample was incubated in the dark at 37 °C for 1 h. The sample was then washed three times (3 min each time) with PBS. The cells were then stained in the dark at room temperature for 10 min before being observed and photographed using a fluorescence-inverted microscope.

### 2.6. Flow Cytometry

Flow cytometry was utilized to detect pyroptosis in cells. The process of incubating and administrating PEDV to cells was the same as that described above for RT-PCR. Then, the cells were digested with EDTA-free trypsin and centrifuged to discard the supernatant. The cells were washed twice with PBS, 100 μL of binding buffer was added, and the cells were resuspended. Next, 5 μL of Annexin V-FITC and 5 μL of PI Staining Solution were added to each sample and incubated for 10 min. Next, 400 μL of binding buffer was added to the sample, which was then mixed thoroughly. The different cell populations were analyzed using a CytoFLEX flow cytometer for flow cytometry analysis.

### 2.7. Statistical Analysis

All experiments were repeated three times or more. All data are expressed as the means ± standard deviation (SD) and were analyzed by the two-tailed Student’s *t*-test followed by Tukey’s multiple-comparison test using Prism software (GraphPad Prism 9.5.1). * *p* < 0.05; ** *p* < 0.01.

## 3. Results

### 3.1. WEPO Attenuated PEDV-Infected Vero Cell Injury

**Figure 1:**
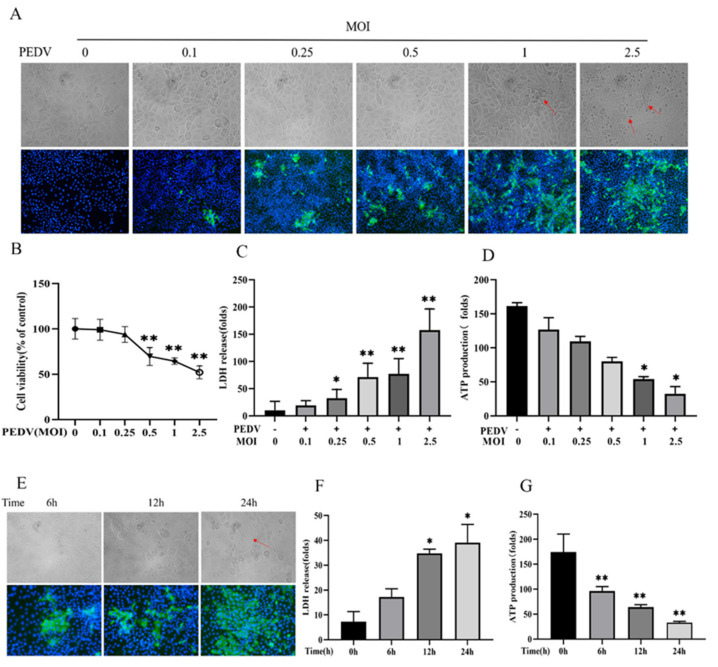
WEPO attenuated PEDV-infected Vero cell injury. (**A**) Morphological observation and immunofluorescence of Vero cells infected with different concentrations of PEDV (The magnification of a microscope is 200 times. The area indicated by the red arrow is the vacuole formed after Vero cell lesion). (**B**) The cytotoxicity of PEDV in Vero cells. Cells were treated with PEDV at an MOI of 0 to 2.5 for 24 h, and cell survival was detected by CCK-8 assay. The data are expressed as the means ± SD (n = 3). ** *p* < 0.01 vs. control. (**C**) The release of LDH was measured after Vero cells were infected with different concentrations of PEDV. The data are expressed as the means ± SD (n = 3). * *p* < 0.05 and ** *p* < 0.01 vs. control. (**D**) The amount of ATP production was measured after Vero cells were infected with different concentrations of PEDV. The data are expressed as the means ± SD (n = 3). * *p* < 0.05 vs. control. (**E**) Morphological observation and immunofluorescence after Vero cells were infected with PEDV at different times (6 h, 12 h, 24 h). (**F**) LDH release was detected after PEDV infection of Vero cells for different periods. The data are expressed as the means ± SD (n = 3). * *p* < 0.05 vs. control. (**G**) The amount of ATP production was measured after PEDV infection of Vero cells at different times. The data are expressed as the means ± SD (n = 3). ** *p* < 0.01 vs. control.

**Figure 2:**
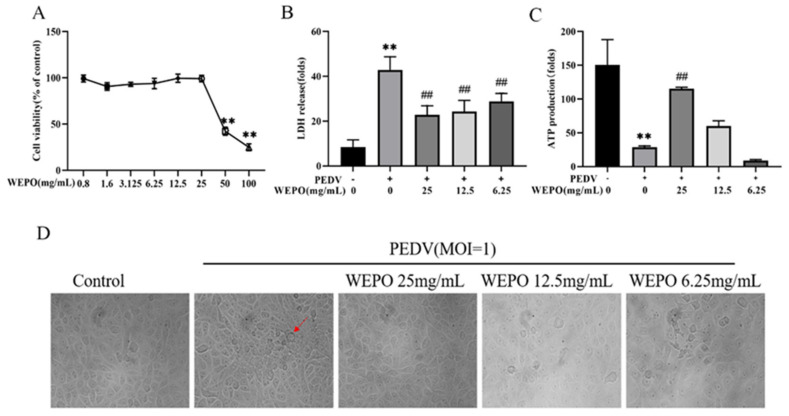
WEPO attenuated PEDV-infected Vero cell injury. (**A**) The cytotoxicity of WEPO on Vero cells. Vero cells were treated with different concentrations of WEPO for 24 h, and the cell survival rate was detected using a CCK-8 assay. The data are expressed as the means ± SD (n = 8). ** *p* < 0.01 vs. control. (**B**) After PEDV infects Vero cells, different concentrations of WEPO were used to detect the release of LDH. The data are expressed as the means ± SD (n = 3). ** *p* < 0.01 vs. control. ## *p* < 0.01 vs. positive control (PEDV). (**C**) PEDV-infected Vero cells were treated with different concentrations of WEPO to detect the amount of ATP production. The data are expressed as the means ± SD (n = 3). ** *p* < 0.01 vs. control. ## *p* < 0.01 vs. positive control (PEDV). (**D**) PEDV-infected Vero cells were treated with different concentrations of WEPO, and cell morphology was observed. (The magnification of a microscope is 200 times. The area indicated by the red arrow is the vacuole formed after Vero cell lesion).

**Figure 3:**
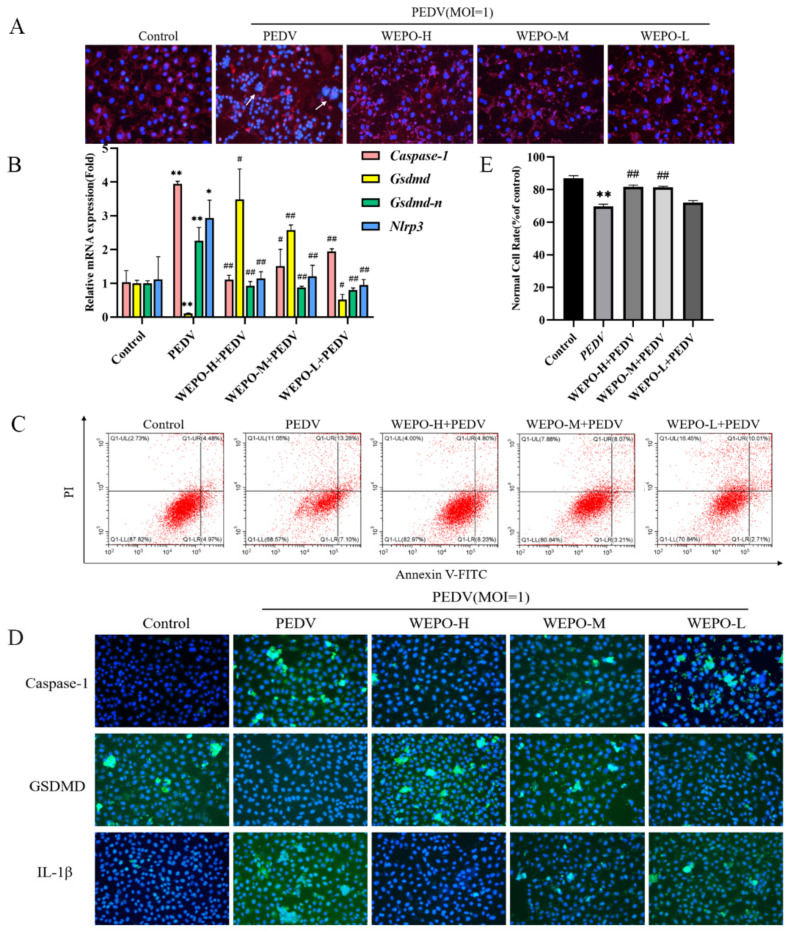
GSDMD participates in the pyroptosis of WEPO-inhibited PEDV-infected Vero cells. (**A**) The integrity of the cell membrane was examined by Dil staining after infection with PEDV and the addition of different concentrations of WEPO. (The magnification of a microscope is 200 times. The white arrow indicates the place where the Vero cells have lesions and rupture, and the cell membrane fuses). (**B**) RT-PCR was used to detect the expression of Caspase-1, GSDMD, GSDMD-N, and Nlrp3 in Vero cells after infection with PEDV and the addition of different concentrations of WEPO. The data are expressed as the means ± SD (n = 3). * *p* < 0.05 and ** *p* < 0.01 vs. control. # *p* < 0.05 and ## *p* < 0.01 vs. positive control (PEDV). (**C**) Detection of the degree of apoptosis in Vero cells by flow cytometry. (**D**) The expression of Caspase-1, GSDMD, and IL-1β in Vero cells was detected by immunofluorescence after infection with PEDV and treatment with different concentrations of WEPO. (The magnification of a microscope is 200 times). (**E**) Flow cytometry data analysis histogram. The data are expressed as the means ± SD (n = 3). ** *p* < 0.01 vs. control. ## *p* < 0.01 vs. positive control (PEDV).

To determine whether WEPO attenuates PEDV-Infected Vero Cell Injury, the CCK-8 assay, LDH release, and ATP synthesis were first examined to evaluate its protective effect in vitro. First, Vero cells were treated with different concentrations (MOI = 0, 0.1, 0.25, 0.5, 1, 5) of PEDV for 24 h, which resulted in dose-dependent inhibition of cytopathy, cell viability, LDH release increase, and ATP synthesis reduction (Figure 1A–D). Additionally, time dependence (6 h, 12 h, and 24 h) was examined (Figure 1E–G), showing that PEDV infection for 24 h was most pronounced. Subsequently, safe concentrations of WEPO were tested, and the results showed that concentrations no higher than 25 mg/mL were not cytotoxic toward Vero cells (Figure 2A). Then, WEPO treatment effectively alleviated PEDV-infected Vero cell damage and death in a dose-dependent manner (6.25, 12.5, and 25 mg/mL), especially at 25 mg/mL, as evidenced by cell viability, LDH release, ATP synthesis, and cell morphology (Figure 2B–D). The immunofluorescence results also showed that WEPO significantly reduced the increase in Caspase-1 and IL-1β induced by PEDV and decreased the expression of GSDMD in a dose-dependent manner (Figure 3D). Given these results, after WEPO pretreatment for 6 h, PEDV infection at an MOI of 1 for 24 h was used in the following experiments in vitro to construct a stable infection model. Accordingly, these results indicated that WEPO could attenuate PEDV-induced Vero cell injury.

### 3.2. WEPO Inhibited Caspase-1/GSDMD-Mediated Pyroptosis in PEDV-Infected Vero Cells

We observed that WEPO plays an important role in enteritis-related diseases by network pharmacology and found that NLRP3, IL-1β, and Caspase-1 are related to pyroptosis in cells [14]. Therefore, we verified whether PO inhibits PEDV-infected Vero cell pyroptosis with cell membrane staining, RT-PCR, immunofluorescence, and flow cytometry to detect pyroptosis marker proteins. Pyroptosis can cause cell swelling and rupture, so we first used DiI staining to detect the integrity of the cell membrane. PEDV infection caused cell rupture and cell membrane fusion (red fluorescence), which was reduced by the addition of WEPO (Figure 3A). Furthermore, RT-PCR was used to assess the expression of related genes in the pyroptosis pathway. As shown in Figure 3B, PEDV treatment significantly induced the activation of pyroptosis-associated gene expression in vitro, including Caspase-1 and IL-1β, while decreasing the expression of GSDMD. As expected, this activation could be reversed by cotreatment with WEPO in a dose-dependent manner. Additionally, the related proteins Caspase-1, IL-1β, and GSDMD were tested by immunofluorescence. These results also showed that the protein levels of Caspase-1 and IL-1β in the PEDV-infected group were significantly higher than those in the control group, while GSDMD was lower. When WEPO was added, compared with those in the PEDV-infected group, the levels of Caspase-1 and IL-1β in the WEPO groups decreased significantly, and the levels of GSDMD increased in a dose-dependent manner (Figure 3D). In addition, to estimate the permeability of the cell membrane, the proportion of cell death was evaluated using Annexin V-FITC staining and flow cytometry (Figure 3C). WEPO significantly reduced PEDV-induced cell death in Vero cells in a dose-dependent manner (Figure 2D), indicating that WEPO pretreatment prevented cell membrane damage attributed to PEDV treatment. These results were also consistent with the network pharmacology results; WEPO could inhibit the changes in Caspase-1/GSDMD caused by PEDV infection, especially at 25 mg/mL.

### 3.3. GSDMD Participates in the Pyroptosis of WEPO-Inhibited PEDV-Infected Vero Cells

**Figure 4:**
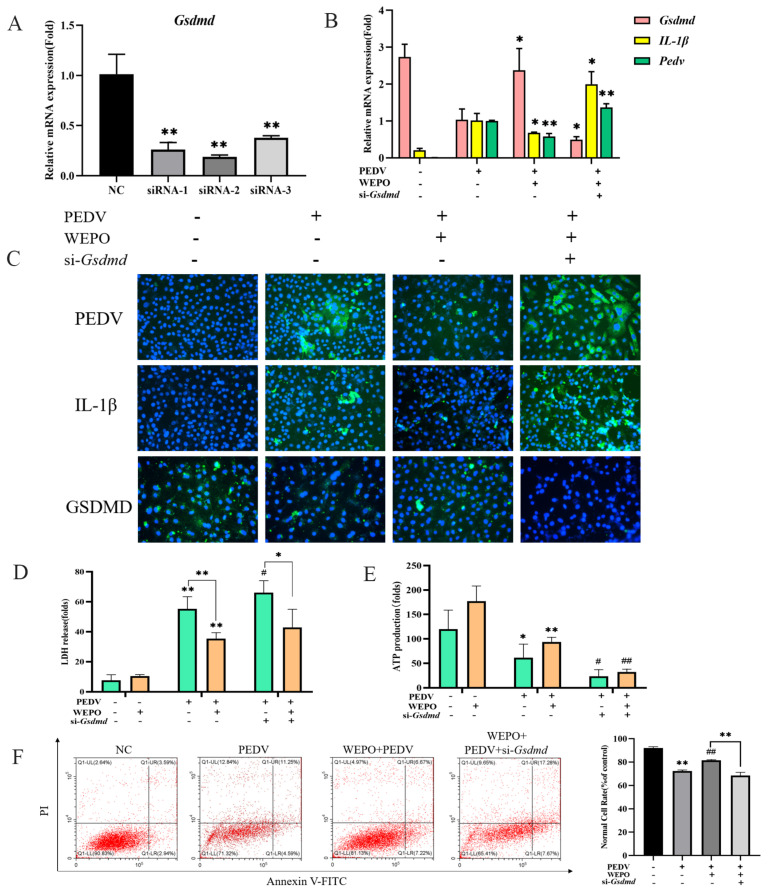
GSDMD participates in the pyroptosis of WEPO-inhibited PEDV-infected Vero cells. (**A**) Detection of the interference efficiency of siGSDMD with RT-PCR. The data are expressed as the means ± SD (n = 3). ** *p* < 0.01 vs. control. (**B**) GSDMD was knocked out, and IL-1β, GSDMD, and PEDV expression was detected through RT-PCR. The data are expressed as the means ± SD (n = 3). * *p* < 0.05 and ** *p* < 0.01 vs. positive control (PEDV + WEPO). (**C**) Expression of PEDV and IL-1β after GSDMD knockout using an immunofluorescence assay. (The magnification of a microscope is 200 times). (**D**) Detection of LDH release after adding PEDV followed by adding purslane and knocking off GSDMD. The data are expressed as the means ± SD (n = 3). * *p* < 0.05. ** *p* < 0.01 vs. control. # *p* < 0.05 vs. positive control (PEDV). (**E**) Detection of ATP generation after adding PEDV followed by adding purslane and knocking off GSDMD. The data are expressed as the means ± SD (n = 3). * *p* < 0.05 and ** *p* < 0.01 vs. control. # *p* < 0.05 and ## *p* < 0.01 vs. positive control (PEDV). (**F**) Detection of apoptosis in Vero cells after knocking out GSDMD with flow cytometry. ** *p* < 0.01 vs. control. ## *p* < 0.01 vs. positive control (PEDV).

Studies have reported that GSDMD is a downstream executor in mediating pyroptosis [15,16]. We observed that the PEDV-infected model increased GSDMD-N protein expression, and WEPO inhibited GSDMD-N protein expression based on the above results (Figure 3B). Therefore, we explored the effects of GSDMD on the pyroptosis of WEPO induced by PEDV infection. We first synthesized GSDMD siRNAs and validated their knockdown efficiency by RT-PCR, which showed that the expression of Gsdmd was significantly reduced (Figure 4A). Then, compared with the control group, the mRNA level of IL-1β induced by PEDV was increased by GSDMD knockdown (Figure 4B). In addition, GSDMD knockout further enhanced the multilabeled fluorescence intensity of PEDV and IL-1β induced by the WEPO model (Figure 4C). After PEDV infection of Vero cells, the release of LDH increased, and the production of ATP decreased. The addition of WEPO alleviated this trend. After GSDMD knockout, the release of LDH induced by PEDV was inhibited and ATP production was increased (Figure 4D,E). Flow cytometry results showed that GSDMD knockout restored the increase in the number of pyroptotic cells induced by PEDV (Figure 4F). These results suggest that GSDMD may be the key factor in WEPO’s inhibition of PEDV proliferation in the Vero cell model.

### 3.4. Caspase-1 Is Needed for GSDMD-N Induction and Pyroptosis of WEPO in Response to PEDV Infection

**Figure 5:**
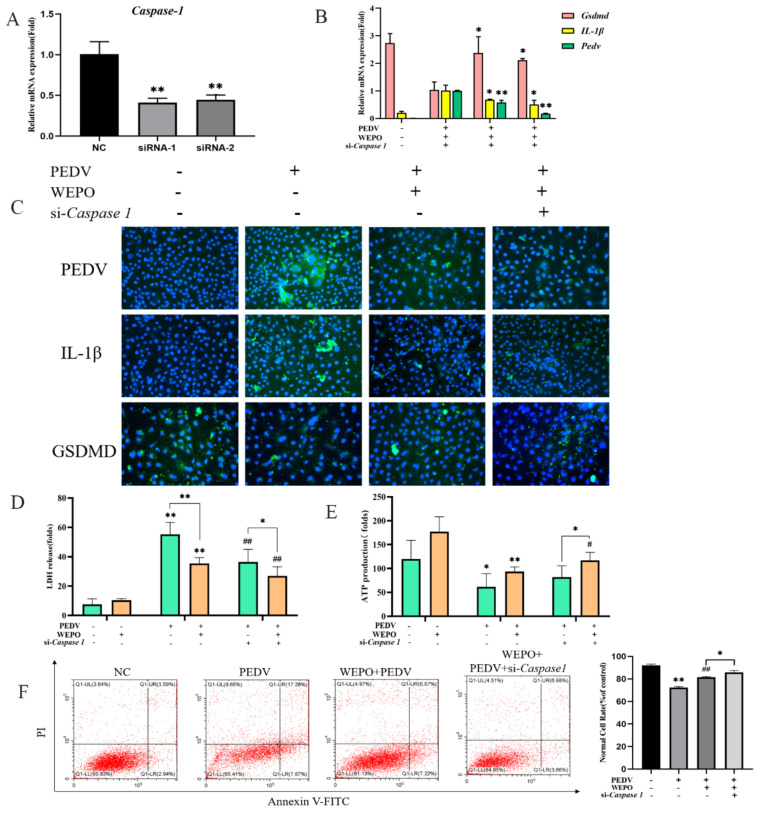
Caspase-1 is needed for GSDMD-N induction and WEPO pyroptosis in response to PEDV infection. (**A**) Detection of the interference efficiency of si-Caspase-1 by RT-PCR. The data are expressed as the means ± SD (n = 3). ** *p* < 0.01 vs. control. (**B**) Caspase-1 was knocked out, and IL-1β, GSDMD, and PEDV expression levels were detected using RT-PCR. The data are expressed as the means ± SD (n = 3). * *p* < 0.05 and ** *p* < 0.01 vs. positive control (PEDV + WEPO). (**C**) Expression of PEDV, GSDMD, and IL-1β after Caspase-1 knockout using an immunofluorescence assay. (The magnification of a microscope is 200 times). (**D**) Detection of LDH release after adding PEDV followed by adding purslane and knocking off Caspase-1. The data are expressed as the means ± SD (n = 3). * *p* < 0.05. ** *p* < 0.01 vs. control. ## *p* < 0.01 vs. positive control (PEDV). (**E**) Detection of ATP generation after adding PEDV followed by adding purslane and knocking out Caspase-1. The data are expressed as the means ± SD (n = 3). * *p* < 0.05 and ** *p* < 0.01 vs. control. # *p* < 0.05 vs. positive control (PEDV). (**F**) Detection of apoptosis in Vero cells after knocking out Caspase-1 by flow cytometry. * *p* < 0.05. ** *p* < 0.01 vs. control. ## *p* < 0.01 vs. positive control (PEDV).

Previous studies have indicated that Caspase-1 is responsible for GSDMD induction in the process of pyroptosis under different stimulation and diverse cellular contexts. In addition, obvious results showed that the protein level of Caspase1 was increased in a sustained manner in PEDV infection, which could be remarkably restored by WEPO (Figure 1C,D). Thus, we further studied the potential role of Caspase-1 in WEPO pyroptosis in PEDV-infected cells. First, Caspase-1 siRNAs were synthesized and validated, which showed that they could be reduced by more than 57% (Figure 5A). Furthermore, WEPO was performed on Caspase-1 siRNAs and control cells. siRNAs were transfected into cells for 24 h to reduce the expression of Caspase-1, and then the effects of Caspase-1 on WEPO-mediated inhibition of pyroptosis in the PEDV infection model were measured using RT-PCR and immunostaining. Compared with the control cells, the expression levels of PEDV and IL-1β induced by WEPO were inhibited by Caspase-1 siRNA (Figure 5B). Moreover, the decreased number of positive cells and the strengthened fluorescence intensity of GSDMD-N and IL-1β induced by the WEPO model were significantly recovered by Caspase-1 siRNA (Figure 5C). After 24 h of transfection of cells with siRNA, WEPO was applied to the cells for 6 h, and then the expression of LDH and ATP was detected. The results showed that the release of LDH decreased and the production of ATP increased after the knockout of Caspase-1 (Figure 5D,E). The results of L-flow cytometry also verify this point (Figure 5F). These results suggest that GSDMD-N induction and WEPO pyroptosis in the Vero cell model were mediated by Caspase-1. In conclusion, the above results showed that the Caspase-1/GSDMD pathway is vital in WEPO-induced PEDV reduction and pyroptosis.

### 3.5. Quercetin, as One of the Main Components of Purslane, Participates in Cell Pyroptosis Mediated by Caspase-1/GSDMD

**Figure 6:**
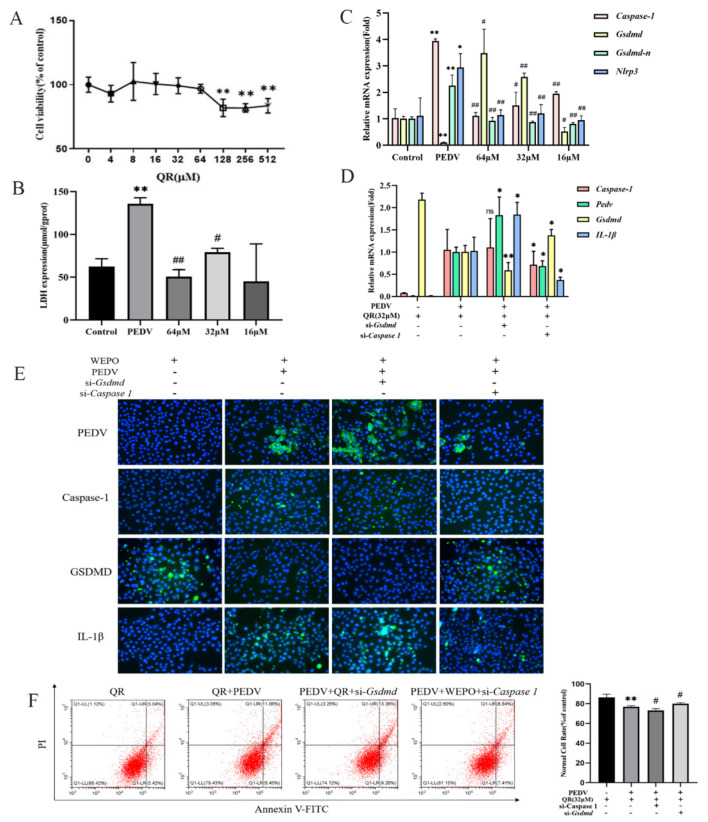
Quercetin, as one of the main components of purslane, participates in cell pyroptosis mediated by Caspase-1/GSDMD. (**A**) The cytotoxicity of QR on Vero cells. Vero cells were treated with different concentrations of QR for 24 h, and the cell survival rate was detected using a CCK-8 assay. ** *p* < 0.01. (**B**) After PEDV infects Vero cells, different concentrations of QR were used to detect the release of LDH. The data are expressed as the means ± SD (n = 3). ** *p* < 0.01 vs. control. # *p* < 0.05 and ## *p* < 0.01 vs. positive control (PEDV). (**C**) RT-PCR was used to detect the expression of Caspase-1, GSDMD, GSDMD-N, and Nlrp3 in Vero cells after infection with PEDV and the addition of different concentrations of QR. The data are expressed as the means ± SD (n = 3). * *p* < 0.05 and ** *p* < 0.01 vs. control. # *p* < 0.05 and ## *p* < 0.01 vs. positive control (PEDV). (**D**) Knockout of GSDMD and Caspase-1, addition of QR at a concentration of 32, and detection of Caspase-1 and IL-1β through RT-PCR. The data are expressed as the means ± SD (n = 3). * *p* < 0.05 and ** *p* < 0.01 vs. positive control (PEDV + QR). (**E**) Immunofluorescence assays were used to detect the expression of PEDV, GSDMD, IL-1β, and Caspase-1 after knocking out GSDMD and Caspase-1. (The magnification of a microscope is 200 times). (**F**) Detection of apoptosis in Vero cells after knocking out GSDMD and Caspase-1 by flow cytometry. ** *p* < 0.01 vs. control. # *p* < 0.05 vs. positive control (PEDV).

Quercetin may play an important role in the anti-inflammatory and antiviral effects of purslane through network pharmacology analysis of the main active components of purslane [17]. Therefore, we speculate that quercetin may inhibit the proliferation of PEDV. First, Vero cells were treated with different concentrations (512, 256, 128, 64, 32, 16, 8, and 4 μM) of quercetin, and the cell survival rate was measured by CCK-8 to screen for safe concentrations of quercetin. The results showed that the safe concentration of quercetin was less than 64 μM (Figure 6A). Based on this, we detected LDH release and the expression of pyroptosis-related genes after infection with PEDV and the addition of different concentrations of quercetin. The results showed that LDH release significantly increased after infection with PEDV, while quercetin significantly reduced LDH release (Figure 6B). At the same time, Nlrp3, Caspase-1, and GSDMD-N significantly increased after infection with PEDV, while GSDMD significantly decreased (Figure 6C). The addition of quercetin significantly improved the increase or decrease in related genes in a dose-dependent manner. The above results indicate that quercetin can inhibit the proliferation of PEDV in Vero cells. In addition, RT-PCR and flow cytometry results showed that after knocking out GSDMD, the increase in IL-1β induced by PEDV was more significant, while knocking out Caspase-1 resulted in an increase in GSDMD and a decrease in IL-1β, significantly inhibiting the degree of PEDV-induced cell apoptosis (Figure 6E,F).

## 4. Discussion

The high incidence rate and mortality of PEDV have brought enormous economic losses to the pig industry, which has attracted people’s attention. In addition, the detection rate has been increasing in recent years [18], suggesting that we need to monitor the variant transmission and prevention of the disease. However, there is currently no specific drug to prevent and treat this disease, which indicates that we need to be vigilant about the spread of this disease and the development of anti-PEDV drugs.

During the screening of anti-PEDV drugs, it was found that the water extract of *Portulaca oleracea* (WEPO) could significantly inhibit the proliferation of PEDV (Figure 2A). The premise of antiviral testing is the analysis of drug cytotoxicity, as drug cytotoxicity may affect the judgment of drug antiviral activity in vitro. In this study, we chose a more sensitive CCK-8 assay to determine the cytotoxicity of WEPO on Vero cells, as these cell types are commonly used in the study of PEDV [19]. We found that WEPO at concentrations of 0.8–25 mg/mL had no cytotoxicity in Vero cells, but when the concentration was 50 mg/mL, cell viability significantly decreased, indicating that the concentration for antiviral testing should be lower than 25 mg/mL. By combining network pharmacology with analysis of the targets of active ingredients in purslane and the pathways related to enteritis [20], we found that genes related to pyroptosis, such as Caspase and IL-1β, play an important role in this process. Then, we verified the network pharmacology results and found that WEPO could improve the viability of PEDV-infected Vero cells, inhibit Caspase-1 and IL-1β expression, and reduce ATP production and LDH release, which are related to pyroptosis. Pyroptosis is a form of cell death manifested by swollen and rounded cells, cell rupture, and the release of mature IL-1β. This phenomenon not only causes damage to normal cells but also produces chemokines that recruit immune cells, which may lead to the spread of the virus [21]. Among them, pyroptosis caused by the Caspase-1/GSDMD pathway could result in cell damage and exacerbate the inflammatory response in the body. GSDMD-mediated pyroptosis is widely involved in various diseases caused by viral invasion into the body. On the one hand, it could dislodge pathogens from the intracellular replication environment and promote phagocytosis and clearance of pathogenic microorganisms by phagocytic and immune cells. On the other hand, excessive inflammation could lead to cascading inflammatory reactions. For example, after infection with the influenza virus, apoptosis is converted to pyroptosis by regulating the type I interferon signaling pathway, promoting the inflammatory response and inhibiting viral replication [22]. Bocavirus induces pyroptosis of respiratory epithelial cells by activating the NLRP3 inflammasome, promoting the release of viral particles, and initiating persistent infection in the body [23]. HIV-1 infection was reported to lead to Caspase-1-mediated pyroptosis in most CD4 T cells [24]. HCV infection triggers caspase-3-mediated cell death and caspase-1-mediated pyroptosis, which is also the main hallmark of pyroptosis [25]. PEDV infection can stimulate cells to activate the NF-κB pathway, activate the inflammasome, exacerbate pyroptosis, and lead to cell damage [26]. In our study, after PEDV infection of Vero cells, the expression of inflammatory factors such as Caspase-1 and IL-1β significantly increased, and the release of LDH significantly increased. The expression of GSDMD significantly decreased, and the production of ATP significantly decreased. Moreover, the cell membrane staining results showed that PEDV infection caused the rupture and fusion of the cell membrane. This indicates that PEDV infection can lead to excessive inflammation in cells, thereby promoting pyroptosis. Therefore, inhibiting GSDMD-induced pyroptosis may be a new target for the treatment of PEDV infection.

The natural products revealed negative or positive effects on different sites of the inflammatory pathway of cellular pyroptosis, suggesting a potential role in the treatment of cellular pyroptosis-related diseases. Studies on pharmacological inhibition of various viral diseases have shown that inhibition of Caspase-1 could block the GSDMD-mediated cell pyroptosis process and reduce the expression levels of IL-1β and IL-18, leading to corresponding therapeutic effects on viral diseases. Disulfiram was found to block cell pyroptosis and cytokine release and then inhibit lipopolysaccharide-induced septic death in mice, further suggesting that drug blockade of pyroptosis plays an important role in the treatment of diseases. In our studies, WEPO inhibited the changes in Caspase1/GSDMD caused by PEDV infection (Figure 2A). We next used siRNA to investigate the role of Caspase-1/GSDMD in WEPO-mediated inhibition of PEDV (Figure 4 and Figure 5). The results showed that knocking down GSDMD significantly increased the viral load of PEDV and that knocking down Caspase-1 affected the inhibitory effect of WEPO on PEDV (Figure 4 and Figure 5). The above results further prove that the Caspase-1/GSDMD pathway is vital in WEPO-induced PEDV reduction and pyroptosis. In addition, pyroptosis mediated by the GSDMD/Caspase-1 pathway may provide potential therapeutic benefits in the pathogenesis of many diseases [15,16,27].

To further explore the main components of the water extract of *Portulaca oleracea*, combined with the results of network pharmacology analysis [28], the effects of kaempferol and quercetin were tested. Previous studies have confirmed that kaempferol and quercetin have significant inhibitory effects on LPS-induced enteritis in mice [29,30]. The addition of quercetin to feed could effectively alleviate the intestinal damage caused by PEDV infection in piglets, improve their intestinal antioxidant capacity, and enhance their lipid metabolism ability in the jejunum [31]. Therefore, we next verified the inhibitory effect of kaempferol and quercetin on PEDV. Unfortunately, kaempferol did not significantly inhibit PEDV proliferation at the cellular level, while quercetin clearly suppressed PEDV proliferation within a safe range, as shown by RT-PCR and immunofluorescence. Then, we examined the effect of quercetin on the Caspase-1/GSDMD pathway and found that it also played an important role in the pyroptosis pathway. In future studies, we will conduct more in-depth research on the effect of quercetin on PEDV. In addition, the flavonoids in purslane may exert antiviral activity by reducing the ROS induced by viral infection through their antioxidant effects, which also warrants further investigation.

## 5. Conclusions

The results showed that PEDV infection caused pyroptosis in Vero cells, while WEPO could inhibit cell drooping caused by PEDV, and showed a dose-dependent effect within a certain range. The Caspase-1/GSDMD pathway played an important role in this process. PEDV is one of the most serious diseases that endanger the pig industry. By analyzing the mechanism of WEPO’s anti-PEDV effect, we lay a foundation for the development of Chinese herbal medicines that can reduce the incidence of PEDV and improve the survival rate of piglets, ensuring the sustainable and healthy development of the pig industry.

## Data Availability

Data are contained within the article.
